# Very Low-Calorie Ketogenic Diet: A Safe and Effective Tool for Weight Loss in Patients with Obesity and Mild Kidney Failure

**DOI:** 10.3390/nu12020333

**Published:** 2020-01-27

**Authors:** Adriano Bruci, Dario Tuccinardi, Rossella Tozzi, Angela Balena, Silvia Santucci, Riccardo Frontani, Stefania Mariani, Sabrina Basciani, Giovanni Spera, Lucio Gnessi, Carla Lubrano, Mikiko Watanabe

**Affiliations:** 1 Nephrology and Dialysis Unit, Santa Maria alla Gruccia Hospital, 52025 Arezzo, Italy; 2 Department of Endocrinology and Diabetes, University Campus Bio-Medico of Rome, 00128 Rome, Italy; 3 Department of Experimental Medicine, Section of Medical Pathophysiology, Food Science and Endocrinology, Sapienza University of Rome, 00161 Rome, Italy

**Keywords:** chronic kidney disease, high protein diet, very low-calorie diet, VLCD, VLCKD, very low energy diet, safety, kidney function, renal function, low carbohydrate diet

## Abstract

Very low-calorie ketogenic diets (VLCKD) are an effective and increasingly used tool for weight loss. Traditionally considered high protein, ketogenic diets are often looked at with concern by clinicians due to the potential harm they pose to kidney function. We herein evaluated the efficacy and safety of a VLCKD in patients with obesity and mild kidney failure. A prospective observational real-life study was conducted on ninety-two patients following a VLCKD for approximately 3 months. Thirty-eight had mild kidney failure and fifty-four had no renal condition and were therefore designated as control. Anthropometric parameters, bioelectrical impedance and biochemistry data were collected before and at the end of the dietary intervention. The average weight loss was nearly 20% of initial weight, with a significant reduction in fat mass. We report an improvement of metabolic parameters and no clinically relevant variation regarding liver and kidney function. Upon stratification based on kidney function, no differences in the efficacy and safety outcomes were found. Interestingly, 27.7% of patients with mild renal failure reported normalization of glomerular filtrate after dietary intervention. We conclude that, when conducted under the supervision of healthcare professionals, a VLCKD is an effective and safe treatment for weight loss in patients with obesity, including those affected by mild kidney failure.

## 1. Introduction

The epidemic of obesity and its comorbidities represents an increasingly worrisome medical and economic burden according to WHO reports [1]. Several strategies are available for weight loss and maintenance, such as the modification of lifestyle (diet and physical activity), pharmacology and surgery [2,3,4,5,6,7,8,9]. Many dietary patterns have been proposed throughout the years, and although several authors tried to determine what was best, it is now acknowledged that there is no optimal choice for each patient both efficacy- and safety-wise, and the treatment should be tailored to the needs, habits, and clinical condition [10].

According to the thrifty gene theory, obesity and its complications are due to the change in food type and availability. In fact, insulin resistance has been linked to the lack of fasting and fullness succession, leading to a reduced ability to safeguard glucose for the most important functions, such as cerebral activity and reproduction [11]. Based on this, dietary interventions mimicking fasting periods have been proposed in order to rescue abilities that were lost throughout the ages. Significantly reduced dietary carbohydrates (less than 50 g/day) lead to ketones synthesis [12]. Although historically linked to diabetic acidosis, ketones may be present in small quantities in many physiological conditions, such as after an overnight fast, subsequent to strenuous physical activity or in response to a protein-rich meal. Ketone bodies are then utilized as fuel by many extra-hepatic tissues, such as the central nervous system, skeletal muscle, and the heart [13]. 

Very Low-Calorie Ketogenic Diets (VLCKDs), dietary interventions falling into the fasting mimicking ones, are characterized by a very low carbohydrate content (<20 g/daily), 1–1.5 g protein/Kg ideal body weight, 15–30 g fat/daily and about 500–800 caloric intake/daily [14]. To favor compliance, VLCKDs are often delivered through meal replacements modelling a Mediterranean diet. Among the advantages of a VLCKD are the rapid weight loss obtained, satiety induction and muscle mass preservation, all of these resulting in increased compliance [15]. VLCKDs are currently recommended as an effective and feasible dietary intervention in subjects with obesity [16]. However, due to the relative abundance of proteins compared to carbohydrates and fats, VLCKDs are often regarded as possibly damaging kidney function, and are usually not recommended in subjects with reduced filtration. 

A systematic review investigating renal outcomes reported that the kidney seems little affected by Very Low Calorie Diets, although the assessed studies only included adults with normal kidney function, and the diets were quite heterogeneous in macronutrient ratio, making results difficult to interpret [17]. Little evidence is instead available relative to the safety profile in patients with kidney function impairment, where the only study investigating a VLCKD by enrolling subjects with normal kidney function together with mild failure did not stratify based on it and therefore reports partial but promising results regarding renal safety [18]. A recent literature revision reported an improvement in renal function parameters upon weight loss in diabetic patients [19]. Taken together, available evidence possibly suggests that a VLCKD, with the profound weight loss usually obtained, might be an effective tool to manage patients with obesity and mild kidney failure.

We therefore herein evaluated the effect of a VLCKD in terms of weight loss, improvement of metabolic syndrome markers and safety outcomes in a population with mild kidney failure and healthy control subjects.

## 2. Materials and Methods 

### 2.1. Study Design and Population 

This was a real life observational prospective study conducted at the High Specialization Centre for the Care of Obesity, Sapienza University of Rome. Patients with obesity accessing the Centre had medical history collected, physical exam and laboratory work performed (hematology, biochemistry) as part of the routine all patients accessing the center undergo for initial evaluation. Those willing to undergo a VLCKD for weight loss purposes were enrolled, so long as they were in absence of contraindications according to national guidelines, such as known hypersensitivity to one or more components used in the meal replacement products; history of cardiac, cerebrovascular, or major gastrointestinal diseases; psychiatric disturbances; diagnosis of insulin-dependent diabetes mellitus (IDDM); pregnancy; lactation; CKD with an estimated glomerular filtration rate (eGFR) <60 [16]. Based on renal function, the patients were stratified in two groups: MCKD (Mild Chronic Kidney Disease) with an eGFR between 60 and 89 mL/min/1.73m^2^, and NKF (Normal Kidney Function) subjects, with an eGFR ≥ 90 mL/min/1.73 m^2^. 

The study protocol was approved by the Medical Ethical Committee of Sapienza University of Rome (ref. CE5475). The study was conducted in accordance with the Declaration of Helsinki (1964) and subsequent amendments. All participants provided written consent before starting their participation in the study.

### 2.2. Diet Protocol

All patients underwent a VLCKD with the use of replacement meals following a protocol consisting in 5 steps (New Penta, Cuneo, Italy). During the first two steps, net carbohydrate intake was set between 20 and 50 g/day. Ketosis was confirmed every week with the use of commercially available urine reagent strips (Ketur-Test, Roche Diagnostics, Switzerland). Protein and lipid intake were approximately 1–1.4 gr/kg of ideal body weight/day and 15–30 g/day, respectively. Recommended water intake was at least 2 lt/day. Total caloric intake was between 450 and 800/day based on calculated ideal body weight. To avoid micronutrients deficiency, mineral and vitamin supplements were recommended throughout the dietary intervention as per current guidelines [16]. During the first step, only meal replacements and a set amount and quality of vegetables were allowed, and during the second step one meal consisted of a protein dish, with one less replacement meal provided. In the subsequent phases, caloric intake gradually increased, and a step-by-step carbohydrate reintroduction was carried out. The mean duration of the whole protocol was 14.9 ± 8.5 weeks, steps 1 and 2 covering the first half and steps 3 to 5 the second half.

### 2.3. Anthropometric Parameters

Body weight and height were obtained in fasting subjects wearing light clothing and no shoes with an empty bladder. The same calibrated scale and stadiometer were used for all patients. Waist circumference was measured in the same instance at the midpoint between the lower rib margin and the iliac crest, the patients had their waist uncovered and were asked to stand with their feet close together and their weight equally distributed on each leg. Systolic and diastolic blood pressure were measured at baseline and at the end of the intervention.

### 2.4. Bioelectrical Impedance Analysis

Bioelectrical Impedance Analysis (BIA) was performed to evaluate indices of body composition (BIO 101 equipment, Akern s.r.l, Pontassieve, Italy): with Fat, Fat Free and Skeletal Muscle Mass (FM, FFM, and MM, respectively) expressed in kg, and Total Body, Intra Cellular and Extra Cellular Water (TBW, ECW and ICW, respectively) expressed in lt. 

### 2.5. Biochemistry 

Blood samples were collected by venipuncture between 8 a.m. and 10 a.m. after an overnight fast. Samples were then transferred to the local laboratory and handled according to the local standards of practice. Insulin, Glucose, Glycosilated Haemoglobin A1C (HbA1C), lipid profile, electrolytes, uric acid, liver enzymes, and renal function were measured. Glomerular Filtration Rate (GFR) was calculated with the Chronic Kidney Disease Epidemiology Collaboration (CKD-EPI) equation, that performs better than other equations at higher GFR, with less bias and greater accuracy, especially when actual GFR is >60 mL/min per 1.73 m^2^ [20]. 

### 2.6. Data Management and Statistical Methods

Data are expressed as mean ± standard deviation (SD) for continuous variables and as percentage for dichotomous variables. Normality was assessed with the Shapiro–Wilk test and variables were Log transformed when the distribution was non-normal. Within-group analysis was performed by dependent sample Student *t*-test. Between group differences were assessed by general linear mixed model analysis of end of diet variables of the NKF and MCKD groups. The variables of group, baseline values, age and gender were included in the model as fixed effects. Differences were considered statistically significant when p < .05. Statistical analysis was performed using GraphPad Prism Version 6.00 for Windows, GraphPad Software, San Diego California USA and SPSS Statistics for Windows, Version 25.0, Armonk, NY, USA: IBM Corp. 

## 3. Results

### 3.1. VLCKD Is Confirmed as a Safe and Effective Tool for Weight Loss and Metabolic Improvement in Subjects with Obesity.

Ninety-two patients with obesity (23 men and 69 women) were consecutively evaluated. The mean age was 51.3 ± 12.2 years; the mean BMI was 33.8 ± 5.8 kg/m^2^. Baseline characteristics are summarized in Table 1. After the dietary intervention, body weight and BMI were significantly decreased, and BIA data showed this was predominantly caused by a significant reduction in fat mass, with a minor yet significant and expected loss in muscle mass. Of note, 1.1% of patients were deemed as non-responders, with <5% weight loss achieved; 11% reached a weight loss between 5% and 10%; the majority of patients lost 10 to 20% initial weight (63.7%), with 24.2% reaching a weight loss equal to or exceeding 20%. A reduction in Total, Intra and Extra cellular water was also observed, consistent with the known diuretic effect of ketogenic diets [21]. Both systolic and diastolic blood pressure improved over time, and 33.3% of those on antihypertensive medication had it reduced or discontinued due to the blood pressure lowering. Glucose metabolism evaluation showed a frank improvement, with a significant decrease in both fasting glycaemia and HbA1c. Lipid metabolism assessment demonstrated a significant reduction in total cholesterol and triglycerides levels, and no significant modification in HDL and LDL levels. Uric acid and ferritin were also significantly decreased (Table 1).

Liver and kidney function evaluation showed no significant changes, other than a small but significant increase in Blood Urea Nitrogen (BUN) levels. The mild but significant dehydration of the subjects at the end of the diet might justify the small increase in albumin and BUN levels. Calcium and phosphate levels showed a slight increase within normal ranges (9.41 ± 0.43 vs. 9.57 ± 0.44 mg/dL, *p*-value < 0.0001; and 3.53 ± 0.51 vs. 3.70 ± 0.46, *p*-value 0.005, respectively), whereas sodium and potassium were stable, as was PTH (Table 1). Finally, no clinical signs of gout, kidney stones or gallbladder disease were reported by patients throughout the dietary intervention, as assessed during follow up visits. 

The following minor adverse events were reported by some patients: constipation, diarrhea, abdominal cramping, nausea, fatigue, hunger, and dizziness, but none were deemed intolerable and, most often, were resolved within the first few days of the dietary intervention. No standardized questionnaire was collected to assess side effects.

### 3.2. Mild Impairment in Kidney Function Does Not Mediate a Difference in Safety and Efficacy Outcomes of a VLCKD

Based on renal function, the patients were stratified into two groups: 38 MCKD (Mild Chronic Kidney Disease) subjects had a glomerular filtration volume (GFV) between 60 and 89 mL/min, corresponding to Stage 2 Chronic Kidney Disease, and 54 NKF (Normal Kidney Function) subjects had a GFV equal to or higher than 90 mL/min. MCKD subjects were then compared to NKF. Unsurprisingly, the MCKD group had a mean age that was higher than that of NKF, but the groups were not different relative to anthropometric parameters other than fat mass, significantly lower in the MCKD, albumin (lower in NKF), and sodium, again lower in NKF. No significant differences in anthropometric parameters (body weight, BMI), blood pressure or biochemical parameters (glucose metabolism and lipid profile, electrolytes, uric acid, hepatic enzymes) were found between the two groups over time (Table 2). Moreover, no between-group difference was observed regarding the percentage of patients reaching 5%, 5–10%, 10–20% or over 20% weight loss. Of note, 27.7% of patients with MCKD reported an improvement of renal function at the end of the dietary intervention leading to an eGFR ≥90.

## 4. Discussion

Management of obesity is of constantly increasing concern nowadays, and chronic kidney disease is a possible complication that may require extra care. Among the available strategies for weight loss and maintenance, VLCKDs are an effective tool, but some concern is present with regard to the treatment of patients with renal failure due to the relative dietary protein excess.

In our real-life observational study, we first assessed the entire enrolled population without taking renal function into account. We report a significant overall weight reduction as expected, with a mean body weight decrease of nearly 20%, and a significant reduction in fat mass (80% of total weight loss) in less than 3 months of dietary treatment, consistent with previous studies [15]. A statistically significant reduction in MM, of little clinical relevance, was observed, and this decrease was paralleled by a reduction in TBW as previously described [21]. The increase diuresis known to happen during a ketogenic diet might explain the TBW finding, that could, in turn, play a role in the BIA assessed MM reduction known to be influenced by body hydration [21]. Both systolic and diastolic blood pressure were reduced as expected. Lipid and glucose metabolism significantly improved, and no safety concern arose.

In fact, hepatic enzymes AST and ALT showed a tendency to decrease, despite not reaching significance, and triglycerides were profoundly decreased, all of which is consistent with reduced intrahepatic triglyceride content and liver size reduction, as previously described in patients with obesity undergoing a VLCKD before bariatric surgery [19,20]. No changes were detected in sodium or potassium levels, suggesting that a VLCKD does not impair the hydroelectrolytic balance. Uric acid was finally significantly decreased, excluding a correlation between VLCKD and hyperuricemia. A recent metanalysis reported an overall neutral effect on uric acid by VLCKDs [22], and a previous study reported similar effect to ours, where a decrease in urate was observed after a VLCKD but not after an LCD [23]. These controversial results might find their explanation in timing and weight loss amplitude, as food groups that typically increase serum uric acid levels are widely consumed in ketogenic diets and might lead to such an effect in the short term [24]. However, it is acknowledged that weight loss is associated with a significant reduction in urate levels [25]. As our patients experienced a mean weight loss of nearly 20% of baseline values, it seems reasonable to conclude that this aspect might have played a predominant role in modulating uric acid levels.

We also observed a significant but slight increase in calcium and phosphorus levels (though remaining in the normal range), that might be attributable to two possible reasons: First, mild dehydration, as observed by TBW reduction, and expected as a result of the significant diuretic effects of VLCKDs, might be responsible for the relative increase in calcium and phosphorus levels due to simple hemoconcentration. The observed elevation in albumin levels point in the same direction. Second, calcium and phosphorus levels could also be increased following bone loss, as it was previously observed in patients on severely calorie-restricted regimens with profound weight reduction [26]. Of note, PTH levels were not altered by the intervention, suggesting that bone metabolism was unaffected, and adequate protein intake and electrolytes supplementation were provided throughout the study, making the latter option less plausible. That being said, as no evidence is currently available regarding change in bone density and quality in patients undergoing a VLCKD regimens, further studies are warranted to look into this safety outcome.

Ferritin levels were also marginally modified by dietary intervention, with a significant reduction over time. As ferritin has been shown to be a marker of inflammation rather than iron deficiency in subjects with obesity [27], and ketosis has been widely proven to have an anti-inflammatory effect [28], we believe that this reduction parallels reduced systemic inflammation in our patients. However, no other inflammatory markers were assessed in this study, and we therefore cannot confirm this hypothesis.

Subjects included in this study were then stratified in two groups based on renal function. No differences between groups were shown in anthropometric parameter changes (body weight, BMI, BIA data) or metabolic profile improvement. Interestingly, a significant proportion of MCKD patients reported a full recovery (eGFR ≥ 90 mL/min/1.73m^2^) of kidney function at the end of the dietary intervention, suggesting that not only is VLCKD an effective and safe weight loss tool in MCKD patients with obesity, but that it also could help ameliorate renal function.

Relative protein excess typical of VLCKDs has been of major concern among clinicians for its kidney-damaging potential, preventing many to propose this intervention to patients with CKD in need of weight loss. In order to assess this safety outcome, creatinine, BUN, eGFR and urinary proteins were evaluated. Creatinine and eGFR were not affected by the dietary intervention and no differences were observed in the between group analysis. Conversely, BUN was slightly increased, most likely as a consequence of increased protein metabolism as previously described [29], with again no difference between the two groups. Current guidelines are inconclusive regarding recommended dietary protein intake in patients with early stages of CKD, with some suggesting .8 g/kg body weight as the optimum [30], and others recommending up to 1.4 g/kg body weight [31]. Recent evidence suggests that the impact of dietary protein on renal function may depend on the protein source, with red meat intake being harmful in a dose dependent manner, and other protein sources such as poultry, fish, egg and dairies not showing such a deleterious effect [32]. Moreover, studies assessing plant-based protein sources (soy and vegetable derived) seem to show that these might even play a renoprotective role [33,34]. VLCKDs’ first steps rely on meal replacements, the protein source of which is whey and plant based, and—when the reintroduction of other protein sources occurs in the subsequent steps—patients are recommended to privilege fish and poultry. Protein intake is never higher than 1.5 g/kg/ideal body weight. It seems therefore reasonable to infer that such a dietary intervention is unlikely to produce any deleterious effect on subjects with stage 2 CKD in the first steps. However, extra caution should be adopted in patients affected by mild kidney disease at all times in three ways. First, these patients should not consume over 1.4 g protein/kg of ideal body weight during all VLCKD steps as per available recommendations [31]; second, protein intake should be carefully monitored during reintroduction phases, where the progressive substitution of meal replacements with protein rich dishes may make patients incur excess protein; third, red meat-derived protein should be strongly discouraged through dietary counselling during the reintroduction phases.

The major strength of our study is the real-life setting and the fact that—to the best of our knowledge—we stratified by renal function for the first time. However, our study also has several limitations. Ketosis was only assessed through urinary excretion of acetoacetate, and no capillary beta-hydroxybutyrate levels were measured due to technical obstacles. Markers of renal function that are unaffected by muscle mass—such as cystatin C—were not assessed, as the real-life setting did not allow so. However, previous evidence shows that creatinine is only very marginally affected by muscle mass [35], and we do not therefore expect a major bias induced by the minor muscle mass reduction the patients experienced during the dietary intervention, making this marker sufficiently reliable. Another limit of this study was that GFR was estimated and 24 h urinary collection was not performed. Finally, for its real-life nature, this study did not comprise a control group, nor it was randomized.

## 5. Conclusions

In conclusion, safety markers including kidney function were unchanged throughout the study and not differentially affected by intervention in the two groups, with efficacy outcomes confirming those of previous studies and—most likely—not depending on kidney function. VLCKD is therefore a safe and effective dietary intervention in patients with obesity affected by mild CKD when conducted under medical supervision in a real-life setting, although caution should be taken in screening for a lack of micronutrients and for altered bone metabolism, as well as in accurately monitoring protein consumption at all times.

## Figures and Tables

**Table 1:** Baseline and end of the diet population characteristics.

	Baseline	End of Diet	*p*
*n*	92	92	
Female gender *n* (%)	69 (65)	69 (65)	
HTN treatment *n* (%)	39 (42.9)		
Age (years)	51.27 ± 12.20		
Weight (Kg)	92.40 ± 18.31	76.82 ± 14.95	**<0.0001**
BMI (kg/m2)	33.85 ± 5.84	28.21 ± 4.90	**<0.0001**
Fat Mass (Kg)	35.63 ± 9.93	24.40 ± 9.00	**<0.0001**
Fat Free Mass (Kg)	56.77 ± 13.40	52.42 ± 10.89	**<0.0001**
Skeletal Muscle Mass (Kg)	37.27 ± 9.57	33.93 ± 8.10	**<0.0001**
TBW (Lt)	42.44 ± 9.85	39.20 ± 8.11	**<0.0001**
ECW (Lt)	19.62 ± 4.47	18.57 ± 3.96	**<0.0001**
ICW (Lt)	22.80 ± 5.79	20.74 ± 4.95	**<0.0001**
SBP (mmHg)	137.1 ± 12.2	132.2 ± 9.2	**<0.0001**
DBP (mmHg)	81.5 ± 6.7	77.4 ± 4.6	**<0.0001**
Creatinine (mg/dL)	0.79 ± 0.17	0.78 ± 0.17	0.139
e GFR (ml/min/1.73m^2^)	94.46 ± 18.75	95.75 ± 18.52	0.32
BUN (g/L)	0.36 ± 0.10	0.39 ± 0.09	**<0.0001**
Glucose (mg/dL)	95.32 ± 13.26	88.25 ± 10.24	**0.002**
HbA1c (%)	5.65 ± 0.81	5.33 ± 0.39	**<0.0001**
AST (mg/dL)	25.38 ± 16.11	20.83 ± 6.12	0.233
ALT (mg/dL)	32.08 ± 26.42	23.44 ± 12.60	0.138
Total cholesterol (mg/dL)	206.91 ± 45.65	184.46 ± 41.17	**0.004**
LDL (mg/dL)	120.17 ± 42.92	117.38 ± 38.65	0.388
HDL (mg/dL)	55.97 ± 18.42	51.69 ± 11.37	0.141
Triglycerides (mg/dL)	156.44 ± 90.87	102.62 ± 35.71	**0.003**
Total Protein (g/dL)	7.07 ± 0.38	7.06 ± 0.39	0.915
Albumin (g/dL)	4.14 ± 0.25	4.18 ± 0.28	**0.035**
Uric Acid (mg/dL)	5.23 ± 1.04	4.83 ± 1.11	**<0.0001**
Calcium (mg/dL)	9.41 ± 0.43	9.57 ± 0.44	**<0.0001**
Phosphorus (mg/dL)	3.53 ± 0.51	3.70 ± 0.46	**0.005**
Sodium (mmol/L)	141.13 ± 2.45	141.51 ± 2.07	0.155
Potassium (mmol/L)	4.53 ± 0.40	4.55 ± 0.40	0.244
PTHi (ng/L)	36.16 ± 10.65	33.66 ± 12.16	0.384
Ferritin (ug/L)	112.34 ± 125.29	101.69 ± 67.72	**0.027**
Urinary protein (mg/dL)	12.43 ± 9.50	11.03 ± 7.15	0.207

Data shown as means ± standard deviation (SD) of the mean. The *p* value is from a dependent sample *t*-test. HTN, hypertension; BMI, Body Mass Index; TBW, Total Body Water; ECW, Extra Cellular Water; ICW, Intra Cellular Water; SBP, Systolic Blood Pressure; DBP, Diastolic Blood Pressure; eGFR, estimated Glomerular Filtration Rate; BUN, Blood Urea Nitrogen; LDL, Low Density Lipoprotein; HDL, High Density Lipoprotein. Significant *p* values are highlighted in bold.

**Table 2:** Differences between NKF and MCKD before and after treatment.

	NKF (e GFR ≥ 90)							MCKD (eGFR 60–89)							
Variable	Baseline			End of Diet			*p*	Baseline			End of Diet			*p*	*p* *
*N*	54						NA	38						NA	
Female gender *n* (%)	43 (80)						NA	22 (68)						NA	0.22
HTN treatment *n* (%)	20 (43.5)						NA	19 (44.2)						NA	
Diet length (weeks)	15.33	±	7.98				NA	15.33	±	7.98				NA	0.645
Age (years)	47.96	±	12.97				NA	55.97	±	9.30				NA	**<0.0001**
Weight (Kg)	94.21	±	18.60	77.94	±	15.67	<0.0001	89.92	±	17.84	75.28	±	13.95	<0.0001	0.510
BMI (kg/m2)	34.46	±	5.69	28.67	±	5.04	<0.0001	33.01	±	6.01	27.56	±	4.68	<0.0001	0.334
Fat Mass (Kg)	37.51	±	9.57	25.93	±	9.45	<0.0001	33.05	±	9.96	22.31	±	7.99	<0.0001	0.742
Fat Free Mass (Kg)	56.69	±	14.10	52.01	±	11.10	<0.0001	56.88	±	12.55	52.97	±	10.72	<0.0001	0.521
Skeletal Muscle Mass (Kg)	37.08	±	10.05	33.58	±	8.14	<0.0001	37.54	±	9.00	34.40	±	8.13	<0.0001	0.542
TBW (Lt)	42.04	±	10.00	38.60	±	7.86	<0.0001	42.98	±	9.76	40.03	±	8.47	<0.0001	0.494
ECW (Lt)	19.53	±	4.49	18.42	±	4.04	<0.0001	19.74	±	4.50	18.78	±	3.90	<0.0001	0.238
ICW (Lt)	22.51	±	5.91	20.37	±	4.78	<0.0001	23.20	±	5.67	21.23	±	5.19	<0.0001	0.561
SBP (mmHg)	136.6	±	14.5	130.9	±	10.8	<0.0001	137.6	±	12.2	132.2	±	9.2	<0.0001	0.998
DBP (mmHg)	82.6	±	7.2	77.3	±	5.2	<0.0001	81.4	±	6.7	77.4	±	4.6	<0.0001	0.841
Creatinine (mg/dL)	0.70	±	0.11	0.71	±	0.12	0.43	0.93	±	0.16	0.88	±	0.17	0.002	0.414
eGFR (ml/min/1.73m^2^)	107.22	±	11.20	105.28	±	14.32	0.263	76.32	±	10.44	82.21	±	15.14	0.002	0.901
BUN (g/L)	0.34	±	0.08	0.38	±	0.07	<0.0001	0.39	±	0.11	0.41	±	0.11	0.052	0.822
Glucose (mg/dL)	96.7	±	14.5	91.73	±	15.23	0.002	91.59	±	11.0	86.60	±	10.02	0.005	0.053
HbA1c (%)	5.68	±	0.96	5.30	±	0.38	<0.0001	5.60	±	0.46	5.39	±	0.40	0.007	0.470
AST (mg/dL)	28.33	±	19.49	22.08	±	6.71	0.218	20.44	±	6.17	19.36	±	5.26	0.732	0.724
ALT (mg/dL)	38.07	±	31.45	26.23	±	15.35	0.15	22.11	±	9.85	20.42	±	8.36	0.759	0.766
Total cholesterol (mg/dL)	206.65	±	48.24	182.61	±	35.54	<0.0001	207.27	±	43.62	187.13	±	49.28	0.148	0.279
LDL (mg/dL)	122.74	±	44.53	121.71	±	30.31	0.233	117.04	±	42.32	113.04	±	46.14	0.958	0.309
HDL (mg/dL)	53.59	±	9.59	51.21	±	8.61	0.491	58.67	±	25.13	52.25	±	14.27	0.199	0.550
Triglycerides (mg/dL)	172.37	±	101.64	99.33	±	30.48	<0.0001	136.27	±	73.48	106.94	±	42.28	0.061	0.249
Total Protein (g/dL)	7.11	±	0.41	7.11	±	0.40	0.48	7.02	±	0.34	7.00	±	0.38	0.352	0.185
Albumin (g/dL)	4.08	±	0.26	4.17	±	0.27	<0.0001	4.21	±	0.21	4.20	±	0.30	0.968	0.226
Uric Acid (mg/dL)	5.06	±	0.98	4.75	±	1.21	<0.0002	5.49	±	1.10	4.96	±	0.94	0.008	0.561
Calcium (mg/dL)	9.41	±	0.46	9.56	±	0.44	<0.0003	9.42	±	0.39	9.59	±	0.44	0.002	0.401
Phosphorus (mg/dL)	3.46	±	0.54	3.67	±	0.49	<0.0004	3.62	±	0.45	3.75	±	0.39	0.168	0.864
Sodium (mmol/L)	140.60	±	2.30	141.16	±	2.02	0.113	141.89	±	2.49	142.00	±	2.06	0.865	0.883
Potassium (mmol/L)	4.48	±	0.43	4.51	±	0.42	0.345	4.59	±	0.35	4.62	±	0.36	0.505	0.920
PTHi (ng/L)	39.04	±	11.89	36.66	±	14.54	0.627	32.33	±	8.16	29.67	±	7.42	0.33	0.528
Ferritin (ug/L)	121.56	±	146.19	90.44	±	67.44	0.238	95.21	±	74.22	116.14	±	67.74	0.036	0.449
Urinary protein (mg/dL)	13.54	±	10.74	11.34	±	7.30	0.157	10.74	±	7.16	10.56	±	6.98	0.882	0.577

Data shown as means ± standard deviation (SD) of the mean. The *p* value is from a dependent sample *t*-test for the within group analysis. The *p* * value shown is from a general linear mixed model analysis of end of diet variables of the NF and MCKD groups for the between groups analysis. The variables of group, baseline values, age and gender were included in the model as fixed effects. For BP, HTN treatment was taken into account. NA, not applicable; NKF, Normal Kidney Function; MCKD, Mild Chronic Kidney Disease; HTN, hypertension; BMI, Body Mass Index; TBW, Total Body Water; ECW Extra Cellular Water; ICW Intra Cellular Water; SBP, Systolic Blood Pressure; DBP, Diastolic Blood Pressure; eGFR, estimated Glomerular Filtration Rate; BUN, Blood Urea Nitrogen; LDL, Low Density Lipoprotein; HDL, High Density Lipoprotein. Significant *p* values are highlighted in bold.
